# Exopolysaccharides directed embellishment of diatoms triggered on plastics and other marine litter

**DOI:** 10.1038/s41598-020-74801-7

**Published:** 2020-10-28

**Authors:** Mohd Jahir Khan, Ramesh Singh, Kunal Shewani, Prashant Shukla, P. V. Bhaskar, Khashti Ballabh Joshi, Vandana Vinayak

**Affiliations:** 1 Diatom Nanoengineering and Metabolism Laboratory (DNM), School of Applied Sciences, Dr. Harisingh Gour Central University, Sagar, Madhya Pradesh 470003 India; 2 Department of Chemistry, School of Chemical Science and Technology, Dr. Harisingh Gour Central University, Sagar, Madhya Pradesh 470003 India; 3 Department of Physics, School of Physical and Mathematical Sciences, Dr. Harisingh Gour Central University, Sagar, Madhya Pradesh 470003 India; 4 National Centre for Polar and Oceanic Research, Vasco Da Gama, Goa, 403804 India

**Keywords:** Environmental sciences, Environmental social sciences, Limnology, Ocean sciences

## Abstract

In the present study, embellishment or beautification of diatoms on substrates like plastics, polydimethylsiloxane, graphite, glass plate, and titanium dioxide, triggered by exopolysaccharides was examined under laboratory conditions. Exopolysaccharides are secreted mainly by primary colonisers, bacteria, which is succeeded by secondary colonisers *i.e.* diatoms. Both diatom (*Nitzschia *sp.*4*) and bacteria (*Bacillus subtilis*) were exposed with substrates separately for 30 days. Diatoms adhere on substrates strongly, not only because of surface roughness of different substrates but also the nanoporous architecture of diatoms which enhanced their embellishment. This study attempted to identify the substrates that adhere to diatoms strongly and was mainly analyzed by scanning electron microscope and further the observations are well supported by math work software (MATLAB). The variation of diatom’s binding on different substrates is due to the influence of marine litters on diatom population in ocean beds where they undergo slow degradation releasing macro, micro and nanoparticles besides radicals and ions causing cell death. Therefore a proof-of-concept model is developed to successfully deliver a message concerning benefit of using different diatom species.

## Introduction

With the widespread use of plastics, the current era is known as Plasticene era^1^. Plastics are more common in human inhabited regions^2–4^ and it is estimated that about 5.25 trillions of plastics as small as 10 mm in size contaminate the oceans^5^.The process of plastic degradation is slow, takes around 300–400 years^6^. It degrades into microplastics and nanoplastics on exposure to UV radiations, waves and mechanical abrasion^7,8^. In addition, many other persistent materials including rubber, metals and glasses are disposed or abandoned in the marine and coastal environments to form marine litter (ML)^9^. These litters tend to sink in water and remain there for long time. Although they break into smaller fragments but are not degraded completely^10^. The litter sinking capability is enhanced by biofilms formed on their surfaces that increases the weight and let them sink in the ocean beds^11^. These biofilms are exopolysaccharides (EPS) produced mainly by bacteria, microalgae and cyanobacteria^12,13^, protists^14,15^, fungi^16,17^and yeasts^18^. The first approachable colonizers on ML are generally bacteria followed by microalgae of which diatoms play a very important role^19,20^. The deposition or adhesion properties of diatoms on the ML is because of EPS produced by diatoms just like in bacteria^21,22^. EPS play important roles in maintaining the structural integrity of biofilms^23^. However, distribution of EPS in different microorganisms may show heterogeneity^24^. Cooksey reported that diatoms embellish themselves on steel and glass after few hours of exposure which follows increased growth due to photosynthetic activity^25,26^. Furthermore, colonization on hydrophobic surfaces is more rapid than the hydrophilic ones. The degree of anchorage depends upon the surface roughness of ML and EPS produced by diatoms and bacteria^27–29^. Biofilming on ML brings diverse changes not only in the ecological niches of marine flora and fauna but also in the environment^30,31^.

Diatoms besides biofilming the ML that enhances the sinking of pollutants in the water bodies fix almost 25% of global CO_2_^32^. They meet 30% of world’s needs for crude oil, due to lipid rich bodies in their cells^33^. Furthermore, diatoms are nature’s freely available silica which has wide applications in forensics, and material science^34–36^. Thus, any change in the diversity of diatoms by ML not only alters both the atmospheric as well as benthic carbon cycle^37,38^ but also change natural reservoir for many high and low value metabolites^39^.

Marine environment is aggressive towards plastics, polymers, glass and metals which are hazardous for marine life^40,41^. Earlier we reported how morphology of diatom, *Gomphonema augur,* showed valve deformation due to presence of trace metal analytes in water^42^. It is well established that different types of environmental pollutants influence diatom cell density, lipid composition and morphology^43^. This study showed that EPS of diatoms and bacteria play an important role in the adherence on ML. The diatom embellishes in different patterns onto ML due to its varied surface roughness and EPS secreted by diatoms either alone or in association with bacteria^44^. In shallow coastal waters, this may result in photochemical hydrolysis of plastics burgeoning into micro and nanoplastics thus disturbing marine ecology including distribution of bacteria and important phytoplanktons like diatoms^45–48^.

## Materials and methods

### Screening of diatom and bacteria for EPS content by FT-IR

Diatoms and bacteria with maximum EPS were selected for adhesion on plastics and other substrates. In order to examine EPS released by different diatom taxon, surface functional groups of closely related species of *Nitzschia sp. 1(NS1), Nitzschia *sp.* 3(NS3), Nitzschia *sp.* 4 (NS4), Pinnularia borealis (PB)* and *Gomphonema parvulum(GP)* were studied. This was firstly done by FT-IR of these diatoms grown at day 1 and 30 in a modified f/2 media^49,50^. In order to grow bacteria environmental water samples was cultured on Luria Bertani agar medium^51^ and different bacteria were identified morphologically and biochemically^52,53^. To select the bacteria with highest EPS, FT-IR of all the bacterial samples was done after 24 h of growth. The method was based on a systematic treatment of FT-IR spectra obtained from dried bacterial and diatom samples^54^. The selected diatom and bacteria were then grown for 30 days on different ML and characterized by FT-IR spectroscopy.

Approximately 1 mg (diatom and bacteria) sample were washed and cleaned with Milli-Q water. The IR spectra of all the test samples were recorded in the range of 400 cm^−1^ to 4000 cm^−1^ using Bruker ATR Alfa II FT-IR spectrometer. Spectra were processed and smoothed using OPUS 7.0 software. Further FTIR spectra of all the samples were normalized and area under various bands (wave-number regions as 1100-1800 cm-1) was calculated using Origin8 software (Origin Lab). The diatom count was carried out using Neubaeur chamber^55^. The correlation values between diatom/bacteria and substrates were generated by MATLAB (Math work) software for SEM images. To study the adhesive character of diatoms and bacteria control of each was taken on 1^st^day and observed via FT-IR and scanning electron microscope (SEM).

### Selection of ML

Five different ML of uniform 1″ × 1″ size having different level of hydrophobicity were prepared (ESI Table S1). Thin film of graphite, polydimethylsiloxane (PDMS) (Sigma-Aldrich, USA) and TiO_2_ (Sigma-Aldrich, USA) was prepared on glass plates of size 1″ × 1″ using spin coater (Spin NXG-P1A, Kolkata, India). TiO_2_ is a component widely used in cosmetic and beauty products^56,57^. Polyethylene is another set of common ML present nearby human habitation and also from sources like industrial waste, shipping nets and ship wreckage^58^. Glass is yet another common cosmopolitan marine litter often dumped in form of glass bottles. The selected ML’s formed two groups: group I, the hydrophilic group, which included glass plates (Gp) and group II, the hydrophobic group, which included PDMS, plastics (pp), graphite (Grp) and TiO_2_^59,60^.

#### Exposure of diatoms and bacteria to ML

Screened diatom and bacteria were exposed to ML and characterized by FT-IR, atomic force microscopy (AFM), SEM and Ellipsometry techniques. About 6800 cell mL^−1^ of the screened diatom having highest EPS content was transferred in 100 mL of f/2 media having different ML. Similarly the screened bacteria were grown for overnight having optical density of ~ 0.6 at 600 nm before exposing them to ML^61,62^.

Diatom and bacterial assemblage and their interaction with ML were studied for 30 days. The variation in EPS functional groups of diatom and bacteria present on different ML was explored by measuring intensity of absorption via IR spectroscopy. Further embellishment of diatoms directed by EPS triggered on substrates was characterized by measuring refractive index using spectroscopy Ellipsometry techniques (J.A. WOOLLAM-2000X, USA). This was further supported by correlation studies from SEM (Nova NanoSEM 450, USA) images at 1st and 30th day of inoculation. The correlation values were calculated from SEM images using MATLAB software (MathWorks). The correlation is defined as

$$\mathrm{Correlation }(\uprho )=\frac{cov\left(x,y\right)}{\mathrm{\sigma x}*\mathrm{\sigma y}}$$

where *cov (x,y)* is covariance between x and y while σ_x _and σ_y_ are the standard deviations of x and y. Further *cov(x,y)* is defined as

$$cov\left(x,y\right)=\frac{\sum \left({x}_{i}-{x}^{o}\right)({y}_{i}-{y}^{o})}{N-1}$$

where x_i_ = data value of x, y_i_ = data value of y and x^o^ and y^o^ are the mean values and N = number of data values.

## Results and discussion

### Screening of diatom and bacteria having maximum EPS

**Figure 1:**
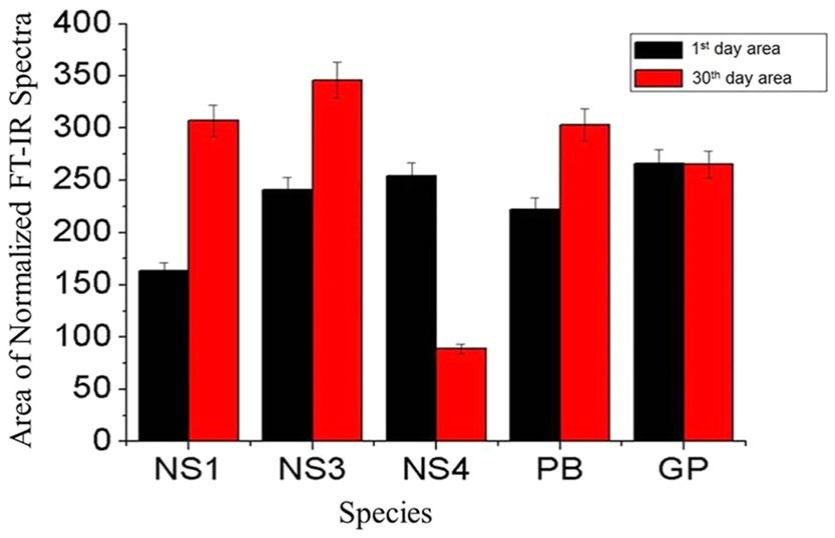
Plot of areas under 1800–1100 cm^−1^ for normalize FT-IR spectra of diatom *NS1, NS3, NS4, PB* and *GP*.

Five diatom cultures *NS1*, *NS3*, *NS4*, *PB* and *GP* were selected for the characterization of polysaccharides, fatty acids (uronic acid) and amide groups. FT-IR characterization of selected diatoms in the range of 1600–1800 cm^−1^ showed the highest area occupied by *NS3* and lowest by *NS4*. However, *NS1, PB* and *GP* were in mediocre range (ESI Table S2). It can be interpreted that the highest content of C=O stretching present in *NS4* (ESI Fig. S1A). However, *NS3* contains the least content of C=O stretching (amide I of protein) as it exhibit higher area value as seen in Fig. 1^63,64^.

Furthermore, diatom growth on 1st and 30th day showed almost similar pattern for various functional groups in all 5 diatom cultures (ESI Fig. S1). Figure 1 showed bands area ranging from 1100 to 1800 cm^−1^of normalized FT-IR spectra for various diatom samples. It showed that there is a variation in the area of diatom spectra for 1st day to 30th day. In most of the samples, the area under 1100–1800 cm^−1^ increased from 1stday to 30thday except for *NS4*, for which it decreased significantly. However, in GP no noticeable change was observed. Hence, diatom *NS4* was chosen for surface interactions and adhesion properties. The FT-IR study of 5 diatoms is coherent with the fact that diatoms cell wall comprise of proteins^65,66^, polyamines^67^ and polysaccharides^68^. However, experimental results have also showed that polysaccharides are far greater than polyamines and proteins in *NS4*^68^.

**Figure 2:**
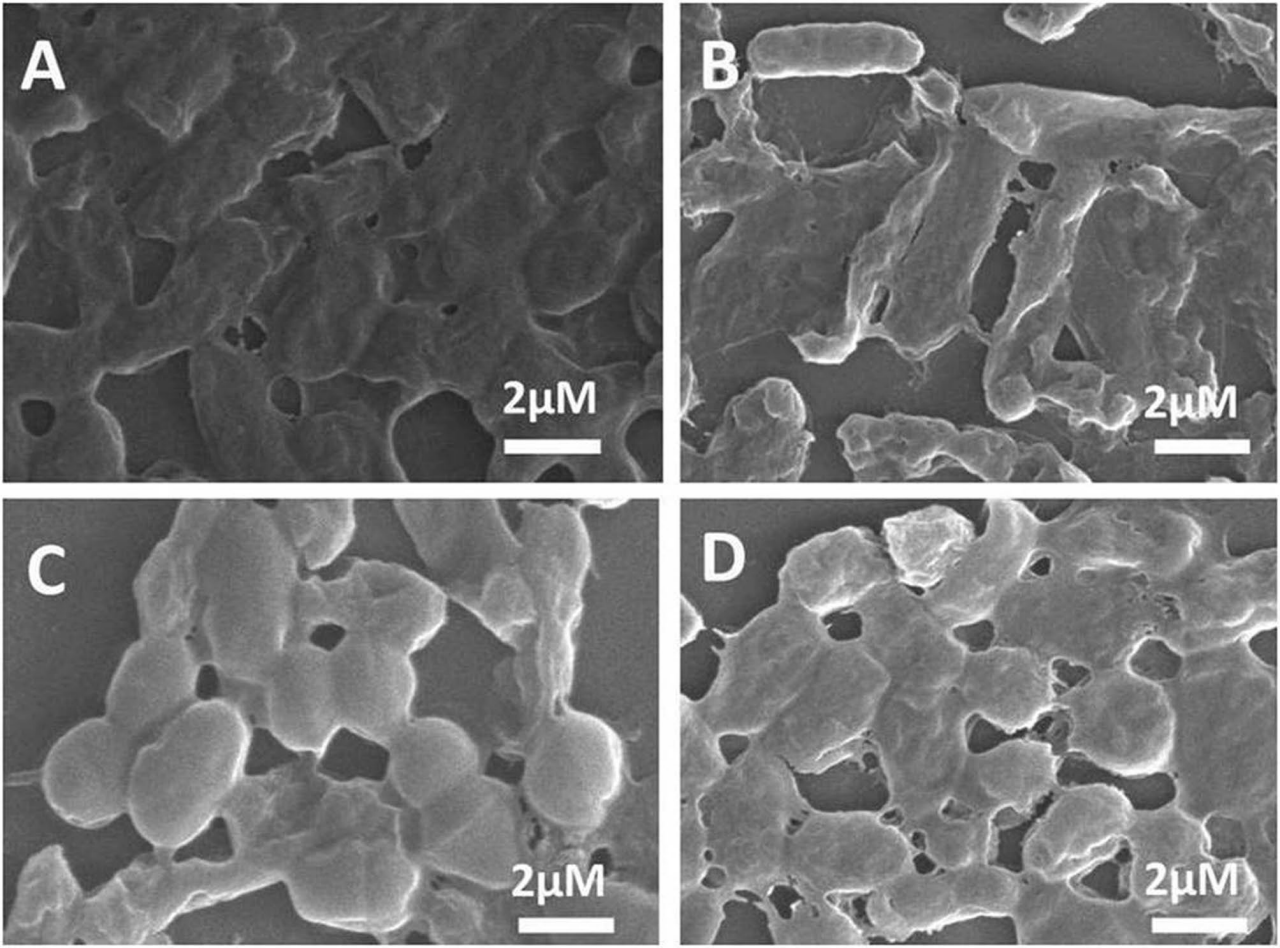
SEM micrographs of (**A**) *Bacillus subtilis*, (**B**) *Enterobacterfaecalis*, (**C**) *Escherchia coli* and (**D**) *Staphylococcus aureus* after 24 h of growth.

The bacteria screened and identified from environmental water samples were of four types viz*; Bacillus subtilis (BS)*, *Enterobacter faecalis (EF)*, *Escherchia coli (EC)* and *Staphylococcus aureus (SA)* as shown in Fig. 2. The FT-IR study showed that polyamines and polysaccharide (1450–1550 cm^−1^) were present in all four bacteria. However, intensity of absorption was lowest in *BS* (ESI Fig. S2A)*.* The low intensity of absorption in *BS* showed that functional groups associated with EPS are not free and therefore poorly exposed. This might be due to robust and viscous biofilm like pattern^69^ which is unlike the mixed coccus type colonies of *EF, EC, SA* as seen in Supplementary Fig. S2B. The high viscosity and sticky surface of *BS* biofilm is because of EPS which play a major role in attachment and anchorage to the substrates.

Thereafter *NS4* and *BS* were incubated with substrates for 30 days to study their adherence on substrates. The control diatom *NS4* and bacteria *BS* showed anchorage among its neighboring cells as seen in their corresponding SEM images (ESI Fig. S3). They actually formed reversible or irreversible adhesions with ML depending upon amount of EPS and the time period of exposure. Hasson and Crowe showed that the attachment of microorganisms with substrate depends on the extracellular polymers produced by microorganisms^70^.

### Characterization of interaction between diatoms and bacteria

**Figure 3:**
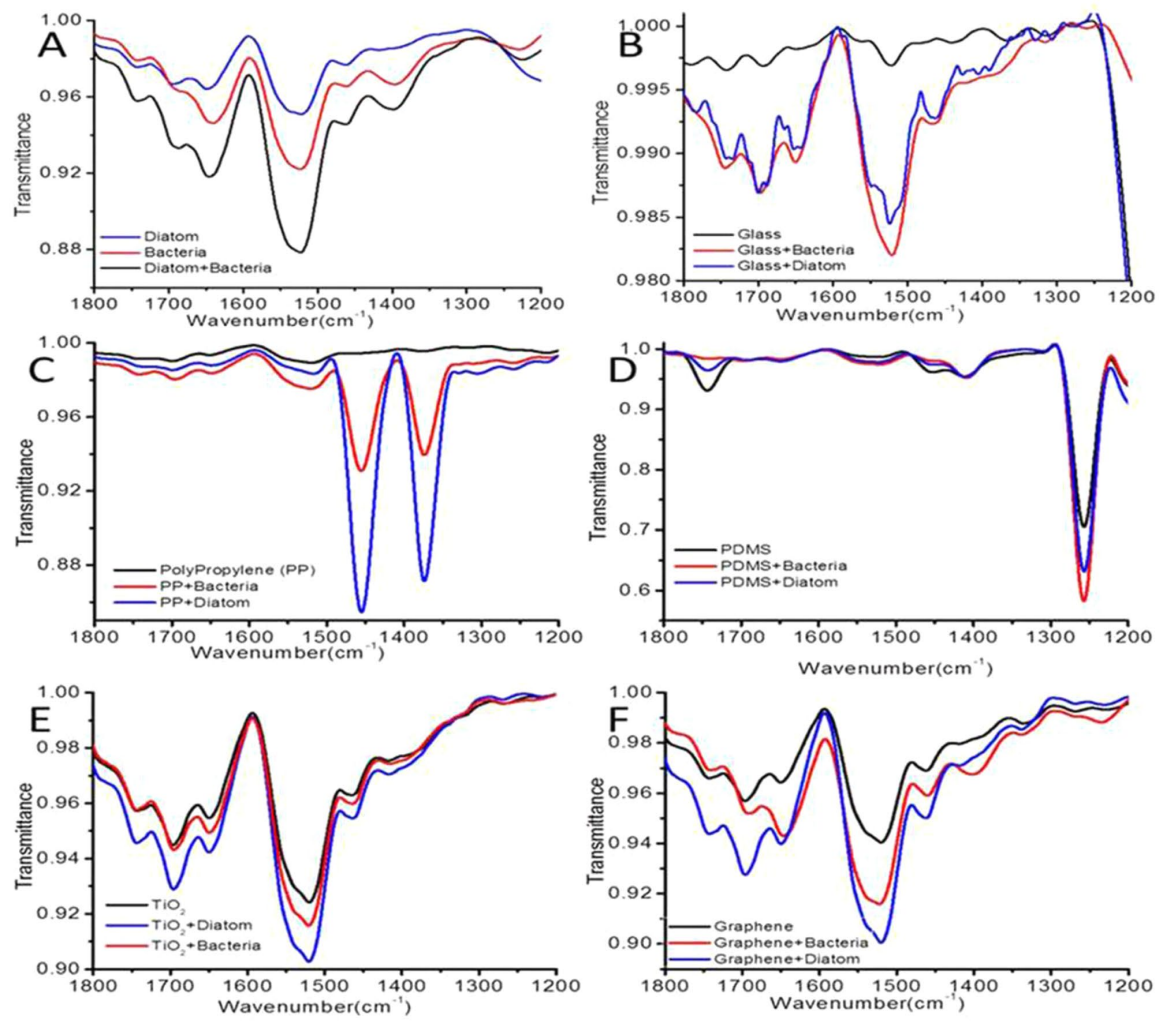
FT-IR spectra in the range of 1100–1800 cm^−1^ shows bacteria and diatoms with ML. (**A**) Control; (**B**) Glass plate; (**C**) Polypropylene; (**D**) PDMS; (**E**) TiO_2_; (**F**) Graphite.

FT-IR was done to characterize the EPS triggered diatom and bacteria embellishment over plastics, PDMS, glass, graphite and TiO_2,_ incubated for 30 days as shown in Fig. 3. Generally EPS is composed of polysaccharides with a certain amount of proteins, lipids and humic substances therefore our study was focused in region between 1200 to 1700 cm^−1^.The control diatom, bacteria and their mixture showed that they do vary in the quantity of EPS even though they have similar polysaccharides groups displayed at around 1500 cm^−1^ (Fig. 3A).The adherence of diatom and bacteria on each of the ML on 30th day showed varied pattern. It was seen that among the ML, PDMS has maximum transmittance due to its crosslinked polymers^71^. It was also found that the hydrophobic surfaces of PDMS displayed more absorption, thus concealing the functional groups of both diatom and bacteria (Fig. 3D). However, at 1250 cm^−1^ region bacteria exhibited more transmittance than diatom. This might be due to presence of cross linkers on PDMS which probably formed covalent bonds not only on EPS secreted by diatom but also got doped inside the nanoarchitectured porous frustules of diatom^72^. This was followed with adherence of diatom and bacteria on plastics (Fig. 3C), graphite (Fig. 3F) and titania (Fig. 3E). However, absorption in glass is not seen due to less hydrophobic surface of glass (Fig. 3B). Off note absorbance of plastics on diatoms was more than bacteria and was quiet distinguishable (Fig. 3C). This also concludes high EPS on cell surface doesn’t allow light to pass through diatom frustules. Hence, diatoms adhere to plastics more strongly than bacteria. The adhesiveness of diatom and bacteria on graphite and TiO_2_ was quiet similar but absorption of diatom was again more than bacteria (Fig. 3E,F). The non-covalent interactions significantly change the key frequency of functional group in FT-IR spectra with change in the ML due to surface roughness^29,73^. The adhesion of diatoms to ML is mainly due to the non-covalent interactions between the functional groups present on the diatom surface and hydrophilic/hydrophobic surface on ML. The monosaccharides present in the cell wall of diatoms and bacteria comprises largely of mannose and glucoronic acid with small amounts of fructose and xylose which may involve in hydrogen bonding and ionic interaction with ML^74–76^.

#### Atomic force microscopy

**Figure 4:**
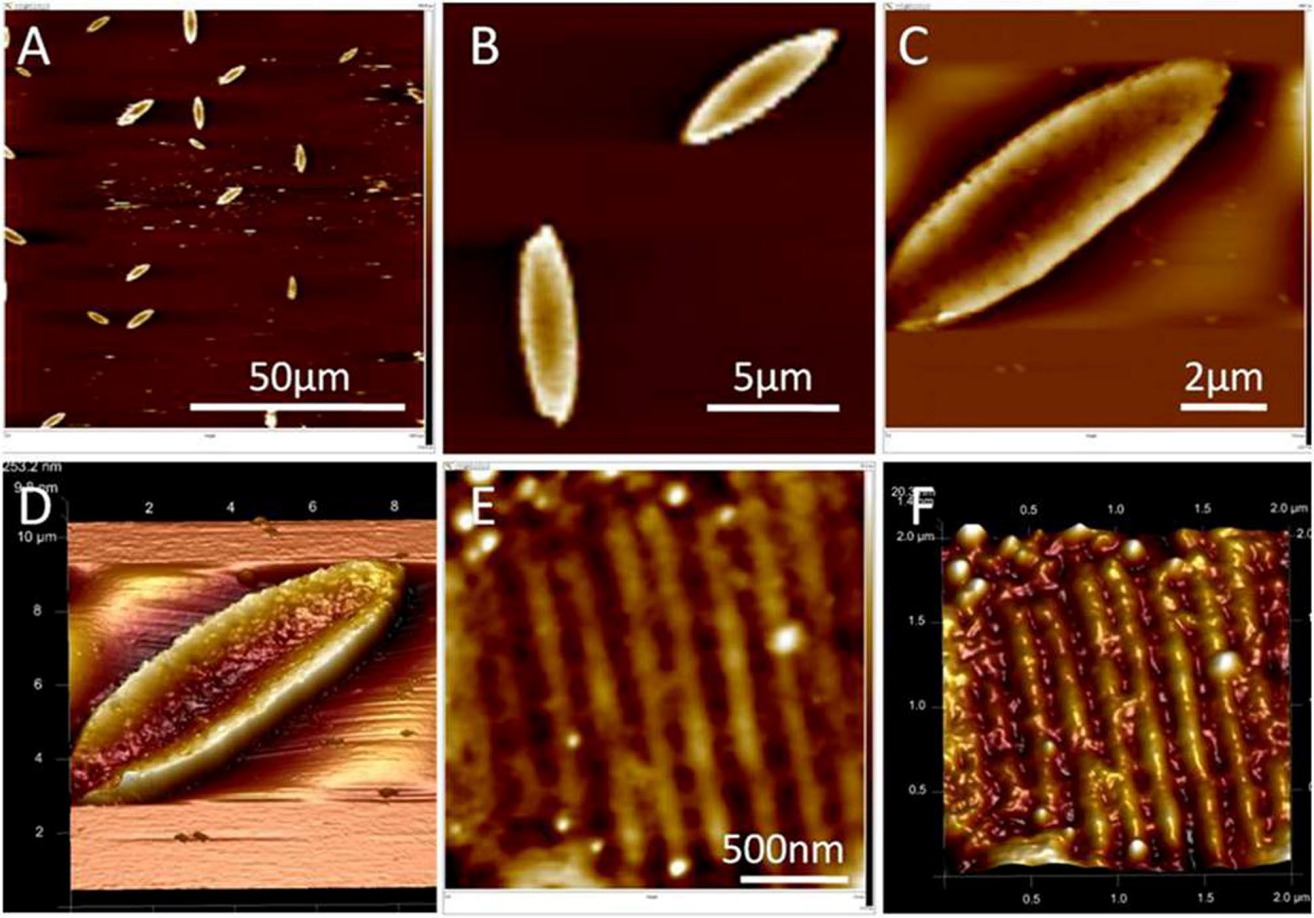
**(A**,**B)** 2D images of discrete intact diatoms positioned over the mica surface, **(C**,**D)** 2D and corresponding high resolution 3D image clearly showing roughness on the diatom surface. Magnified 2D (**E**) and corresponding 3D (**F**) view of filled pores of diatom frustules.

Surface composition of ML plays an important role in the attachment and dominance of primary (bacteria) and secondary (diatoms) colonizers. The uneven distribution of EPS along the diatom cell surface allowed different ML to adhere at varying degrees depending upon their hydrophobicity or hydrophilicity. Further, the surface roughness of diatom is unique due to its nanoporous silica. The ridges and furrows demonstrate the nanoporous architecture of diatoms which further enhance their anchorage on the ML (ESI Fig. S3). This is well demonstrated by AFM studies of *NS4* (Fig. 4). Figure 4A,B show AFM images of diatom *NS4* on a hydrophilic inert mica surface. High resolution AFM images clearly showed roughness on the diatom surface (Fig. 4C,D). However, Fig. 4E,F showed raised humps and valleys due to uneven distribution of EPS on the well arranged porous structure frustules.

AFM image of bacteria *BS* is comparatively smooth with little or no indentations as showed in ESI Fig. S4. Therefore, nanoporous architecture of diatoms exhibit strong embellishments of EPS directed diatoms on ML^77^. It is important to add that the major biomass on these ML is created by diatoms and not bacteria^25^.On the contrary there have been fewer studies on adherence of diatoms on different ML compared to bacteria. We therefore further extended our studies by analyzing the correlation studies created by SEM images of diatoms on these substrates. This was followed by studying the measurement of refractive index via Ellipsometery spectroscopy.

### Study of interfacial surface chemistry of NS4 on ML via SEM, correlation and ellipsometery studies

**Figure 5:**
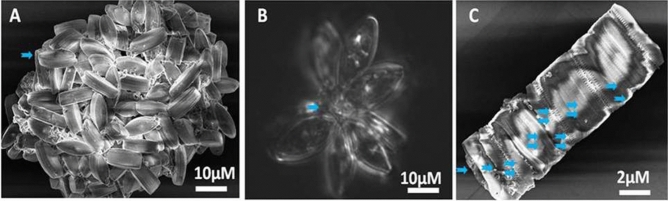
Diatom *NS4* grown for 30 days on glass (A&B) and on plastic substrate(C).

The screened diatom *NS4* was studied for its EPS triggered assemblage on different substrates under SEM. *NS4* EPS formed a floral bouquet anchoring on a glass surface due to less hydrophilicity of glass surface and EPS on diatom surfaces (Fig. 5A,B). Figure 5C showed SEM image of *NS4* stacked monolayer on a plastic substrate. This is the most common assemblage due to two wall structure of diatoms (hypotheca and epitheca)^78^.

**Figure 6:**
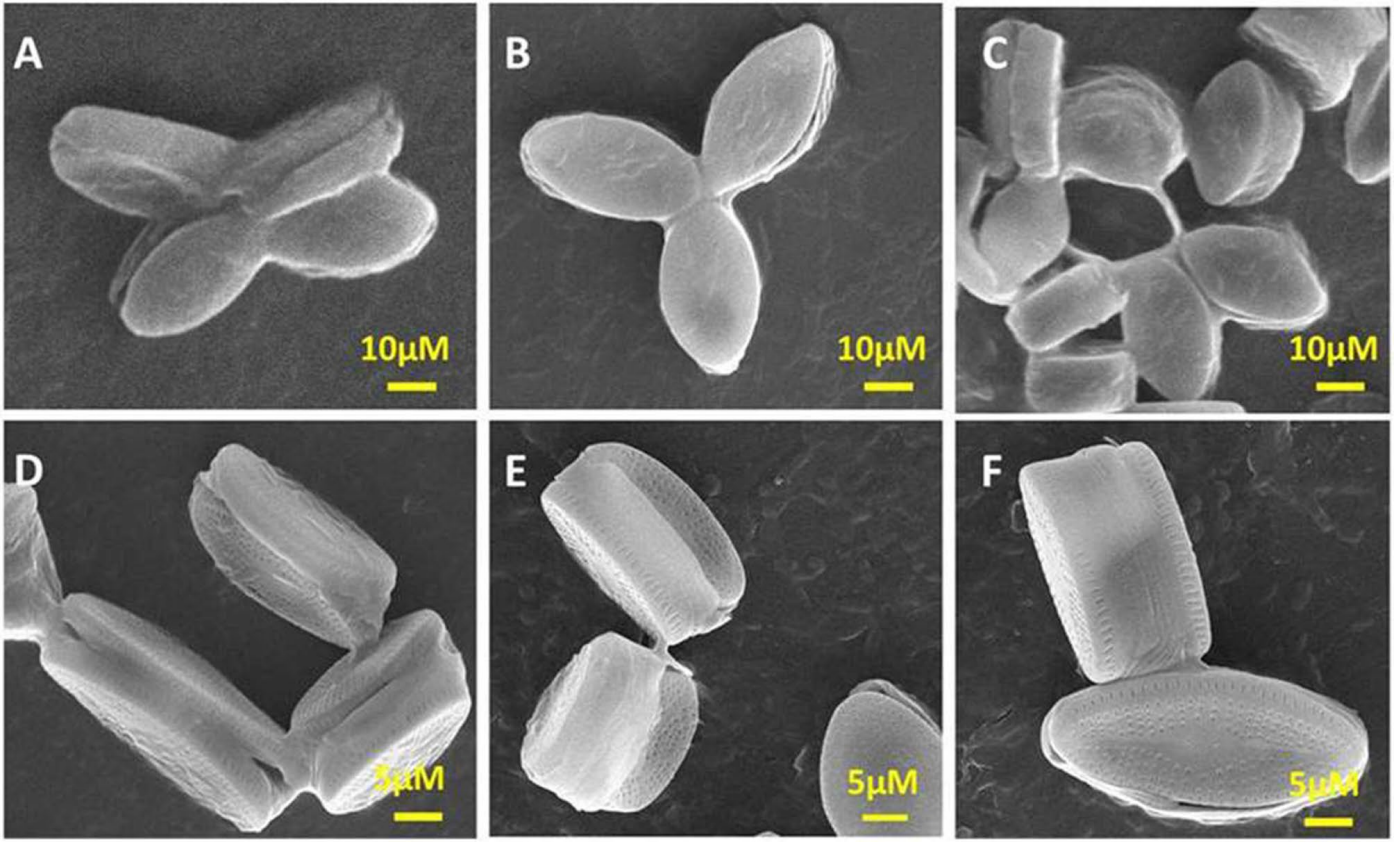
Diatoms on PDMS (**A**–**C**) and plastic surfaces (**D**–**F**) for 30 days.

ESI Figs. S5–S9 further showed SEM images of diatoms embellishment on the surface of glass, graphite, PDMS, plastic, and TiO_2_ substrates. Diatoms were arranged in singlet, doublet, floret, bunch or in stack. *NS4* arranged in florets of 3 or 4 when exposed to PDMS for 30 days can be seen in Fig. 6A–C. However, with plastics it forms stacked and floral bouquets (Fig. 6D–F). Glass plate triggered formation of diatoms in bunches (Fig. 5A,B and ESI Fig. S5) as compared to florets or stacked assembled organization of diatoms in hydrophobic ML like graphite (ESI Fig. S6), PDMS (ESI Fig. S7) and plastics (Fig. 5C and ESI Fig. S8). This justified the hydrophilic/hydrophobic properties and surface roughness of different substrates. In contrast, on titania surface (TiO_2_), diatoms stacked normally one upon another or in any singlet or doublet arrangement (ESI Fig. S9). PDMS showed most adhesive template and formed diatom trifoliate or tetrafoliate leaf like structures (ESI Fig. S7; Fig. 6A–C). However, on plastic surfaces diatoms stacked showing anchorage point at the tip of their frustules (Fig. 6D–F and ESI Fig. S8).

The organization of diatoms on different substrates may vary for different ML or debris. Therefore we had individually studied diatom *NS4* and its adherence properties on different substrates (plastics, PDMS, titania, graphite and glass). The adherence of diatoms on these substrates in different patterns was further characterized by correlation studies.

#### Correlation study between diatoms and different ML

**Figure 7:**
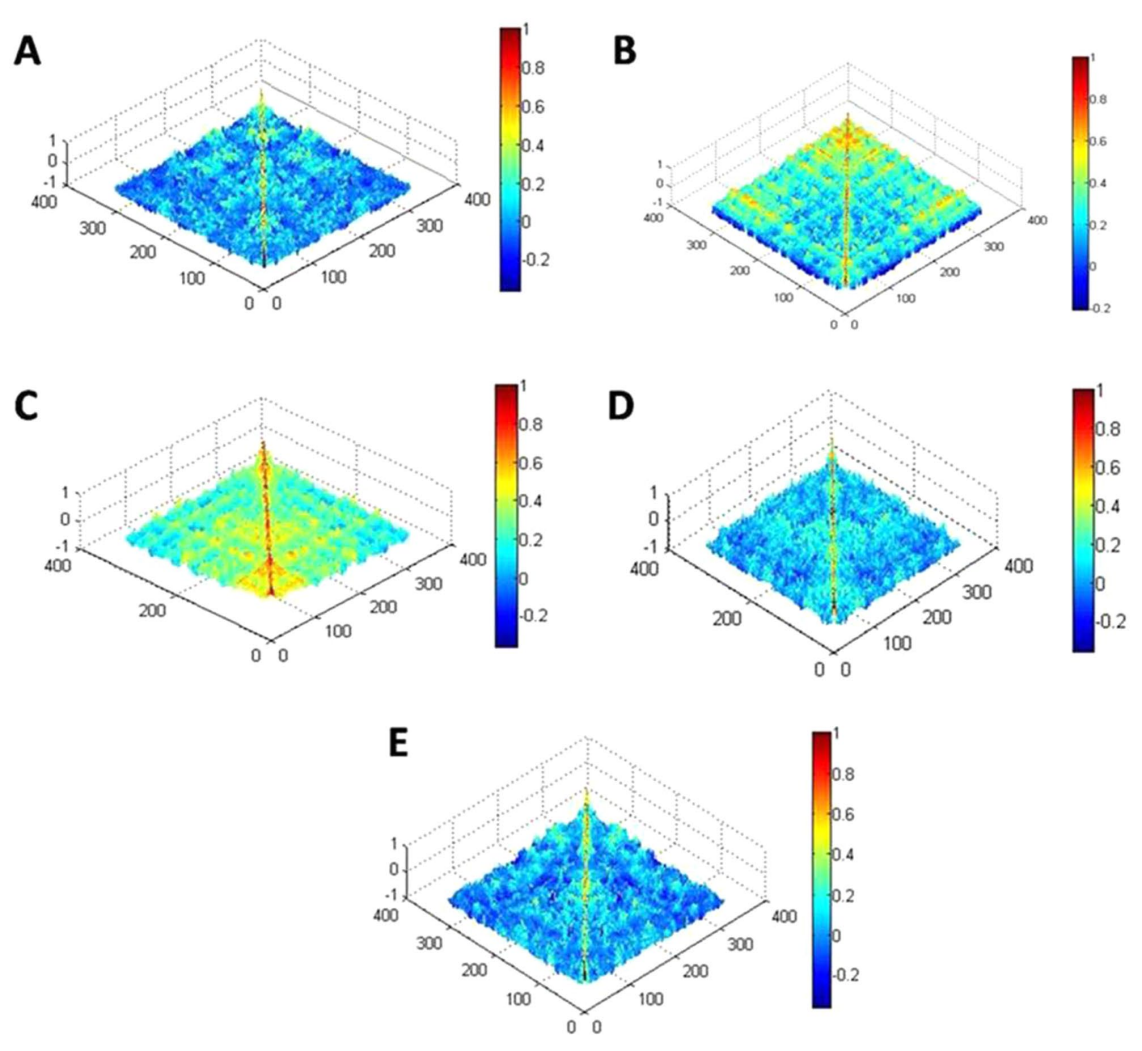
Covariance matrix plot of SEM images of diatoms embellished on different ML; (**A**), covariance matrix plot of Glass, (**B**) Graphite; (**C**) PDMS, (**D**) Plastic and (**E**) TiO_2_. The covariance matrices show the correlation between the components present in the pixels of the images; the blue color represents the smallest one while red shows the largest one.

Figure 7 shows correlation values derived from SEM images of diatom film on various substrates using MATLAB software. It can be seen that there is variation in the color as we move from one image to other for different ML. Further, in these images the value of correlation coefficient is shown by colors. On the color scale, minimum value is represented by blue and maximum by red color. The value of correlation increased as we move from blue to red side. It can be perceived from Fig. 7 that the correlation was highest for PDMS and lowest for graphite in the entire region of image. The variations in the correlation for different substrate reflected the magnitude of adhesive force between substrate material and diatom films. Reduction in the correlation confirms the decrease in the adhesive force between substrate and sample. This decrease in adhesive force influenced the deposition of diatom film on various substrates. It can be seen from Fig. 7 that the adhesive force between PDMS and plastics with diatoms is larger than the adhesive force between diatoms and any other substrates (glass, PDMS, TiO_2_ and graphite).

The mean value of correlation which is quantified from SEM images of diatom samples on different substrates are shown in Table 1. It was found that the mean correlation value was highest for PDMS (0.19), followed by plastics (0.04), graphite (0.037), TiO_2_ (0.03), and least for glass (0.02). However, there is small variation in the correlation values for other materials. This large value confirms the strong binding between the diatoms and substrate. The main cause of binding was the adhesive force between substrate and diatoms. Moreover, the last column in Table 1 and ESI Fig. S10 showed the ratio of correlation for various substrates with respect to glass substrate. It can be seen that the ratio was significantly high; roughly 8 times for PDMS that again confirmed the strong adhesive force for PDMS material than glass.

**Table 1:** Mean correlation values of diatoms enmeshed on different substrates.

S. No	Name of substrate	Mean correlation value for 100 µm	Ratio w.r.t. glass plate
1	Glass plate	0.02	1
2	PDMS	0.19	8.48
3	Plastics	0.04	1.75
4	Graphite	0.037	1.21
5	TiO_2_	0.03	1.30

#### Ellipsometric study of interaction of diatoms on different ML

**Figure 8:**
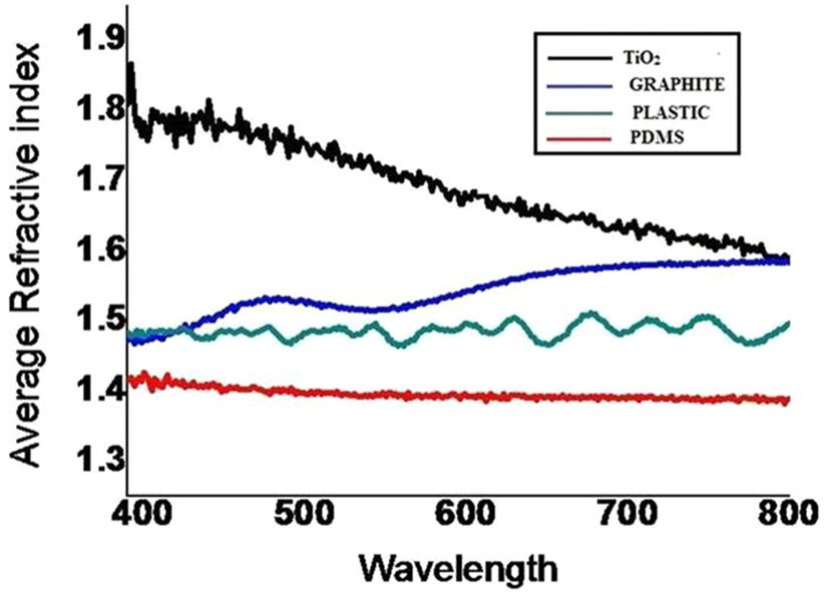
Average refractive index for diatom exposed to glass, grapheme, PDMS and TiO_2_ for 30 days.

Different ML substrates embellished with diatom (*NS4*) were studied by ellipsometric techniques on 30th day of exposure. Figure 8 showed the variation of refractive index for diatom grown on different substrates. It can be seen that different substrate materials influence the refractive index (RI). The value of average refractive index with respect to glass is lowest for PDMS (1.4 ± 0.01), followed by plastics (1.48 ± 0.34) and graphite (1.58 ± 0.91) and highest TiO_2_ (1.66 ± 0.44) over the entire visible region. This variation in the refractive index may arise because of the roughness and adhesive force between diatoms and substrate. Furthermore, it was observed that the refractive index and correlation values have inverse relation. The largest correlation of PDMS had lowest refractive index while graphite exhibited largest refractive index and lowest correlation value. So as correlation increases, the index of refraction decreases. This confirmed that refractive index decreases as the adhesive force increases.

Our results are in concordance with findings from Carson et al. who examined 17 trawls for the presence of microorganism using SEM and FT-IR^79^. Among 83 items which were examined and identified using FT-IR, 59% were polyethylene, 33% were polypropylene and 8% were polystyrene. The biofilming observed on these plastic items were that of *Bacillus* bacteria (mean 1664 ± 243 individuals mm^2^) and Pennate diatoms (1097 ± 154 mm^2^). It was found that centric diatoms were found in very low densities (9 ± 6 mm^2^) with coccoid bacteria being less (169 ± 39 mm^2^). In another study of diatom biofilming on steel by cell surface and cell–cell interaction enhanced by its nanoscale structure and EPS on its surface had been demonstrated by various techniques including FTIR, SEM, correlation and AFM^80^. Besides this EPS played an important role in aggregating the diatoms sinking the ML to benthic beds. The interactions between EPS and ML via reversible or irreversible chemical bonds played a crucial role in degradation of ML. This also affected the diversity of diatoms or bacterial communities in the phycosphere around EPS zone on these ML.

Michels et al. tested whether micro plastics found on the surface of water are involved in the aggregation of natural particles and if biofilms formed on the surface of micro plastics enhance the aggregation potential of these micro plastics^81^. The natural biogenic community tested by them mainly constituted diatoms and dinoflagellates, *Ceratium.* It was found that stickiness is the key factor in the aggregation of biofilms which is mainly due to EPS and DNA secreted by diatoms and other planktons. The microplastics form aggregates with the natural biogenic particles in the sea water but as the diatom biofilm is formed on the surface of microplastics the aggregation is further enhanced. Thus our work showed that the colonization of micropalstics with biofilms increasing their potential to aggregate with biogenic particles. The incorporation of diatom biofilm enhanced the sinking rate as compared to aggregation with other plankton biofilms making effective sinking of ML at different rates. The embellishment of diatoms on different ML tells us that though EPS plays a crucial role but surface roughness of litter is an important factor to know how diatoms adhered to them. Litter in due course of time degrades due to photolysis and influence of other environmental factors thus releasing macro, micro and naoparticles, free radicals which lyses not only the diatom community but also may bring fatal changes in both its morphological and molecular diversity^82^. On the other hand these embellishments may also save them from harsh environments and delay the degradation of these litters which accumulates marine garbage harmful for marine life.

## Conclusions

The comparative study of diatoms and bacteria on different substrates (plastics, PDMS, TiO_2_, graphite and glass) belonging to hydrophilic and hydrophobic groups showed that diatoms adhered strongly to hydrophobic substrates like PDMS and plastics compared to glass. The EPS from both diatoms and bacteria showed presence of common polysaccharide and uronic acids. The correlation values for diatoms incubated with substrates were highest for PDMS (0.19) followed by plastic which indicated that the surface roughness of PDMS and plastics was greater than that for TiO_2_ and graphite. These results were further supported by ellipsometery data which showed that the average refractive index with respect to glass for diatoms was lowest for PDMS and the highest TiO_2_ over the entire visible region. Thus the EPS secreted by diatoms (*Nitzschiasp.4*) form strong anchorage with hydrophobic substrates like PDMS and plastics. Indeed they seem to act as saviors for marine environment by delaying the degradation ML into micro and nanoparticles. However, in this course of time these ML especially plastics undergo photolysis releasing particles, ions and free radicals which either lyse a particular community or bring changes in their genome. This possibly changes the diatom diversity and succession. If disposal of ML is not controlled it may alter the diatom density in marine waters.

## Supplementary information

Supplementary Information.

## References

[1] Stager C (2011). Deep Future: The Next 100,000 Years of Life on Earth.

[2] Browne MA (2011). Accumulation of microplastic on shorelines woldwide: sources and sinks. Environ. Sci. Technol..

[3] Depledge MH (2013). Plastic litter in the sea. Mar. Environ. Res..

[4] Desforges J-PW, Galbraith M, Dangerfield N, Ross PS (2014). Widespread distribution of microplastics in subsurface seawater in the NE Pacific Ocean. Mar. Pollut. Bull..

[5] Shim WJ, Thomposon RC (2015). Microplastics in the ocean. Arch. Environ. Contam. Toxicol..

[6] Sharuddin SDA, Abnisa F, Daud WMAW, Aroua MK (2016). A review on pyrolysis of plastic wastes. Energy Convers. Manag.

[7] Ter Halle A (2017). Nanoplastic in the North Atlantic subtropical gyre. Environ. Sci. Technol..

[8] Ivleva NP, Wiesheu AC, Niessner R (2017). Microplastic in aquatic ecosystems. Angew. Chem. Int. Ed..

[9] Adler, E. *UNEP/IOC Guidelines on Survey and Monitoring of Marine Litter*. 186, Reprint at https://wedocs.unep.org/xmlui/ (2009).

[10] Yang X, Sun L, Xiang J, Hu S, Su S (2013). Pyrolysis and dehalogenation of plastics from waste electrical and electronic equipment (WEEE): A review. Waste Manage..

[11] Xu X (2019). Marine microplastic-associated bacterial community succession in response to geography, exposure time, and plastic type in China's coastal seawaters. Mar. Pollut. Bull..

[12] Parikh A, Madamwar D (2006). Partial characterization of extracellular polysaccharides from cyanobacteria. Bioresour. Technol..

[13] Boonchai R, Kaewsuk J, Seo G (2015). Effect of nutrient starvation on nutrient uptake and extracellular polymeric substance for microalgae cultivation and separation. Desalin. Water Treat..

[14] Jain R, Raghukumar S, Tharanathan R, Bhosle NB (2005). Extracellular polysaccharide production by thraustochytridprotists. Mar. Biotechnol..

[15] Lee Chang KJ (2014). Comparison of *Thraustochytrids aurantiochytrium* sp., *Schizochytrium* sp., *Thraustochytrium* sp., and *Ulkenia* sp. for production of biodiesel, long-chain omega-3 oils, and exopolysaccharide. Mar. Biotechnol..

[16] Hwang HJ, Kim SW, Xu CP, Choi JW, Yun JW (2004). Morphological and rheological properties of the three different species of *Basidiomycetesphellinus* in submerged cultures. J. Appl. Microbiol..

[17] Elisashvili VI, Kachlishvili ET, Wasser SP (2009). Carbon and nitrogen source effects on Basidiomycetesexopolysaccharide production. Appl. Biochem. Microbiol..

[18] Pavlova K, Grigorova D (1999). Production and properties of exopolysaccharide by *Rhodotorulaacheniorum* MC. Food Res. Int..

[19] Patil JS, Anil AC (2005). Quantification of diatoms in biofilms: standardisation of methods. Biofouling.

[20] Cooksey K, Wigglesworth-Cooksey B (1995). Adhesion of bacteria and diatoms to surfaces in the sea: a review. Aquat. Microb. Ecol.

[21] Liu H, Fang HH (2002). Extraction of extracellular polymeric substances (EPS) of sludges. J. Biotechnol..

[22] Nichols CM, Guezennec J, Bowman J (2005). Bacterial exopolysaccharides from extreme marine environments with special consideration of the southern ocean, sea ice, and deep-sea hydrothermal vents: a review. Mar. Biotechnol..

[23] Xiong Y, Liu Y (2013). Importance of extracellular proteins in maintaining structural integrity of aerobic granules. Colloids Surf..

[24] Stewart PS, Franklin MJ (2008). Physiological heterogeneity in biofilms. Nat. Rev. Microbiol..

[25] Cooksey B, Costlow JD, Tipper RC (1984). The Attachment of Microfouling Diatoms.

[26] Riebesell U (2000). Unicellular C4 photosynthesis in a marine diatom. Nature.

[27] Fletcher M, Loeb G (1979). Influence of substratum characteristics on the attachment of a marine pseudomonad to solid surfaces. Appl. Environ. Microbiol..

[28] Sweat LH, Johnson KB (2013). The effects of fine-scale substratum roughness on diatom community structure in estuarine biofilms. Biofouling.

[29] Fazey FM, Ryan PG (2016). Biofouling on buoyant marine plastics: An experimental study into the effect of size on surface longevity. Environ. Pollut..

[30] Winder M, Reuter JE, Schladow SG (2008). Lake warming favours small-sized planktonic diatom species. Proc. Natl. Acad. Sci. USA.

[31] Sun J (2011). Effects of changing pCO2 and phosphate availability on domoic acid production and physiology of the marine harmful bloom diatom Pseudo-nitzschiamultiseries. Limnol. Oceanogr..

[32] Gordon R, Losic D, Tiffany MA, Nagy SS, Sterrenburg FA (2009). The Glass Menagerie: diatoms for novel applications in nanotechnology. Trends Biotechnol..

[33] Krebs W, Gladenkov A, Jones G (1999). Diatoms in oil and gas exploration. The Diatoms: Applications for the Environmental and Earth Sciences.

[34] Vinayak V, Joshi KB, Gordon R, Schoefs B (2017). Nanoengineering of diatom surfaces for emerging applications. Diatom Nanotechnology.

[35] Ghobara MM, Seckbach J, Gordon R (2019). On light and diatoms: A photonics and photobiology review. Diatoms: Fundamentals and Applications.

[36] Vinayak V, Gautam S (2019). Diatoms in Forensics: A Molecular Approach to Diatom Testing in Forensic Science. Diatoms: Fundamentals and Applications.

[37] Yanko V, Arnold AJ, Parker WC (1999). Effects of marine pollution on benthic foraminifera. Modern Foraminifera.

[38] Doney SC, Fabry VJ, Feely RA, Kleypas JA (2009). Ocean acidification: the other CO2 problem. Annu. Rev. Mar. Sci..

[39] Vinayak V (2015). Diatom milking: a review and new approaches. Mar. Drugs.

[40] Worm B, Lotze HK, Jubinville I, Wilcox C, Jambeck J (2017). Plastic as a persistent marine pollutant. Annu. Rev. Env. Resour..

[41] da Costa JP, Santos PS, Duarte AC, Rocha-Santos T (2016). (Nano) plastics in the environment-sources, fates and effects. Sci. Total Environ..

[42] Gautam S, Pandey L, Vinayak V, Arya A (2016). Morphological and physiological alterations in the diatom *Gomphonemapseudoaugur* due to heavy metal stress. Ecol. Indic..

[43] Ahirwar A, Gupta S, Kashyap M, Shukla P, Vinayak V (2019). Differential cell viability in *Nitzschiapalea* on exposure to different organic and inorganic environmental effluents. Int. J. Environ. Sci. Technol..

[44] Grossart HP, Levold F, Allgaier M, Simon M, Brinkhoff T (2005). Marine diatom species harbour distinct bacterial communities. Environ. Microbiol..

[45] Nenadović T (2015). Development of periphytic diatoms on different artificial substrates in the Eastern Adriatic Sea. Acta Bot. Croat..

[46] Browne MA, Galloway TS, Thompson RC (2010). Spatial patterns of plastic debris along estuarine shorelines. Environ. Sci. Technol..

[47] Lusher AL, Burke A, O’Connor I, Officer R (2014). Microplastic pollution in the Northeast Atlantic Ocean: validated and opportunistic sampling. Mar. Pollut. Bull..

[48] Yonkos LT, Friedel EA, Perez-Reyes AC, Ghosal S, Arthur CD (2014). Microplastics in four estuarine rivers in the Chesapeake Bay, USA. Environ. Sci. Technol..

[49] Vinayak V, Gordon R, Gautam S, Rai A (2014). Discovery of a diatom that oozes oil. Adv. Sci. Lett..

[50] Guillard RR, Ryther JH (1962). Studies of marine planktonic diatoms: I. *Cyclotella nana*Hustedt, and *Detonulaconfervacea* (Cleve) Gran. Can. J. Microbiol..

[51] Sezonov G, Joseleau-Petit D, d'Ari R (2007). Escherichia coli physiology in Luria-Bertani broth. J. Bacteriol..

[52] Carlone GM, Valadez MJ, Pickett MJ (1982). Methods for distinguishing gram-positive from gram-negative bacteria. J. Clin. Microbiol..

[53] Mac Faddin JF (1976). Biochemical Tests for Identification of Medical Bacteria.

[54] Marcotte L, Kegelaer G, Sandt C, Barbeau J, Lafleur M (2007). An alternative infrared spectroscopy assay for the quantification of polysaccharides in bacterial samples. Anal. Biochem..

[55] Guillard RR, Sieracki MS, Andersen RA (2005). Counting cells in cultures with the light microscope. Algal Culturing Techniques.

[56] Ni S (2018). Innovations upon antioxidant capacity evaluation for cosmetics: A photoelectrochemical sensor exploitation based on N-doped graphene/TiO_2_nanocomposite. Sens. Actuators.

[57] Srinivas K (2016). The current role of nanomaterials in cosmetics. J. Chem. Pharm. Res..

[58] Galgani F, Hanke G, Maes T (2015). Global distribution, composition and abundance of marine litter. Marine Anthropogenic Litter.

[59] Shent H, Pugh R, Forssberg E (1999). A review of plastics waste recycling and the flotation of plastics. Resour. Conserv. Recycl..

[60] Groszek A (1970). Selective adsorption at graphite/hydrocarbon interfaces. Proc. R. Soc..

[61] Koch AL (1970). Turbidity measurements of bacterial cultures in some available commercial instruments. Anal. Biochem..

[62] Singh R, Kumar Mishra N, Kumar V, Vinayak V, Ballabh Joshi K (2018). Transition metal ion-mediated tyrosine-based short-peptide amphiphile nanostructures inhibit bacterial growth. ChemBioChem.

[63] Fernando I (2017). FTIR characterization and antioxidant activity of water soluble crude polysaccharides of Sri Lankan marine algae. Algae.

[64] Kumar V (2018). Fast Fourier infrared spectroscopy to characterize the biochemical composition in diatoms. J. Biosci..

[65] Hecky R, Mopper K, Kilham P, Degens E (1973). The amino acid and sugar composition of diatom cell-walls. Mar. Biol..

[66] Tesson B (2008). Contribution of multi-nuclear solid state NMR to the characterization of the *Thalassiosirapseudonana* diatom cell wall. Anal. Bioanal. Chem..

[67] Pohnert G (2002). Biomineralization in diatoms mediated through peptide-and polyamine-assisted condensation of silica. Angew. Chem. Int. Ed..

[68] Myklestad SM (1995). Release of extracellular products by phytoplankton with special emphasis on polysaccharides. Sci. Total Environ..

[69] Seminara A (2012). Osmotic spreading of *Bacillus subtilis* biofilms driven by an extracellular matrix. Proc. Natl. Acad. Sci. USA.

[70] Hasson DF, Crowe CR (2013). Materials for Marine Systems and Structures: Treatise on Materials Science and Technology.

[71] Adam M, Lairez D, Karpasas M, Gottlieb M (1997). Static and dynamic properties of cross-linked poly (dimethylsiloxane) pregel clusters. Macromolecules.

[72] Costello CM, Yeung CL, Rawson FJ, Mendes PM (2012). Application of nanotechnology to control bacterial adhesion and patterning on material surfaces. J. Exp. Nanosci..

[73] Webb HK, Crawford RJ, Sawabe T, Ivanova EP (2008). Poly (ethylene terephthalate) polymer surfaces as a substrate for bacterial attachment and biofilm formation. Microbes Environ..

[74] Handa N (1969). Carbohydrate metabolism in the marine diatom *Skeletonemacostatum*. Mar. Biol..

[75] Tesson B, Hildebrand M (2013). Characterization and localization of insoluble organic matrices associated with diatom cell walls: insight into their roles during cell wall formation. PLoS ONE.

[76] Wilkinson J (1958). The extracellular polysaccharides of bacteria. Bacteriol. Rev..

[77] Higgins MJ, Molino P, Mulvaney P, Wetherbee R (2003). The structure and nanomechanical properties of the adhesive mucilage that mediates diatom substratum adhesion and motility. J. Phycol..

[78] Laney SR, Olson RJ, Sosik HM (2012). Diatoms favor their younger daughters. Limnol. Oceanogr..

[79] Carson HS, Nerheim MS, Carroll KA, Eriksen M (2013). The plastic-associated microorganisms of the North Pacific Gyre. Mar. Pollut. Bull..

[80] Landoulsi J, Cooksey K, Dupres V (2011). Review–interactions between diatoms and stainless steel: focus on biofouling and biocorrosion. Biofouling.

[81] Michels J, Stippkugel A, Lenz M, Wirtz K, Engel A (2018). Rapid aggregation of biofilm-covered microplastics with marine biogenic particles. Proc. Natl. Acad. Sci. USA.

[82] deCarvalho CC (2018). Marine biofilms: a successful microbial strategy with economic implications. Front. Mar. Sci..

