# Supplementary material for: Exopolysaccharides directed embellishment of diatoms triggered on plastics and other marine litter
Source: Sci Rep. 2020 Oct 28;10:18448. doi: 10.1038/s41598-020-74801-7 (PMC7595185; doi:10.1038/s41598-020-74801-7)
Supplement: Supplementary file 1 — Supplementary Information. [file 41598_2020_74801_MOESM1_ESM.doc]

**ESI**

**Exopolysaccharides directed embellishment of diatoms** **triggered** **on plastics and other marine litter**

Mohd Jahir Khan^1^, Ramesh Singh^2^, Kunal Shewani^3^, Prashant Shukla^3*^, P.V Bhaskar^4^, Khashti Ballabh Joshi^2*^, and VandanaVinayak^1^*****

**^1*^**Diatom Nanoengineering and Metabolism Laboratory (DNM), School of Applied Sciences, Dr. Harisingh Gour Central University, Sagar, Madhya Pradesh 470003, India.

Email*: kapilvinayak@gmail.com; vvinayak@dhsgsu.ac.in

**^2^**Department of Chemistry, School of Chemical Science and Technology**,** Dr. Harisingh Gour Central University, Sagar, Madhya Pradesh 470003, India. 470003.

**^3^**Department of Physics, School of Physical and Mathematical sciences**,** Dr. Harisingh Gour Central University, Sagar, Madhya Pradesh 470003, India.

**^4^**National Centre for Polar and Oceanic Research, Vasco Da Gama, Goa, 403804, India.

**Table S1. Literature reported surface wettability of substrates used in this study is given in the following table.**

| **S.No.** | **Materials/**  **Substrates** | **Contact Angles** | **References** |
| --- | --- | --- | --- |
| 1 | Glass | 6.7° | Wei, M., Bowman, R. S., Wilson, J. L. & Morrow, N. R. *J. Colloid Interface Sci.* **157**, 154-159 (1993). |
| 2 | Polypropylene | 121.5° | Masaeli, E., Morshed, M. & Tavanai, H. *Surf. Interface Anal.* **39**, 770-774 (2007). |
| 3 | TiO_2_ | 50-70° | Stevens, N., Priest, C. I., Sedev, R. & Ralston, J. *Langmuir* **19**, 3272-3275 (2003). |
| 4 | Graphite | 95-100° | Taherian, F., Marcon, V., van der Vegt, N. F. A. & Leroy, F. *Langmuir* **29**, 1457-1465 (2013). |
| 5 | PDMS Membrane | 130-160° | Zhu, P., Kong, T., Tang, X. & Wang, L. *Nat. Commun.* **8**, 15823, (2017).  Khan, M. J., Singh, R., Joshi, K. B. & Vandana V. *RSC Adv.* **9**, 22410-2241 (2019). |

**Table S2.** Peak areas covered in the range of 1100-1800 cm^-1^ for normalize FT-IR spectra of diatom NS1, NS3, NS4, PB and GP on day 1 and 30.

| **Species** | **1^st^ day Area** | **30^th^ day Area** |
| --- | --- | --- |
| NS1 | 164 | 308 |
| NS3 | 241 | 347 |
| NS4 | 254 | 89 |
| PB | 222 | 303 |
| GP | 266 | 265 |

**
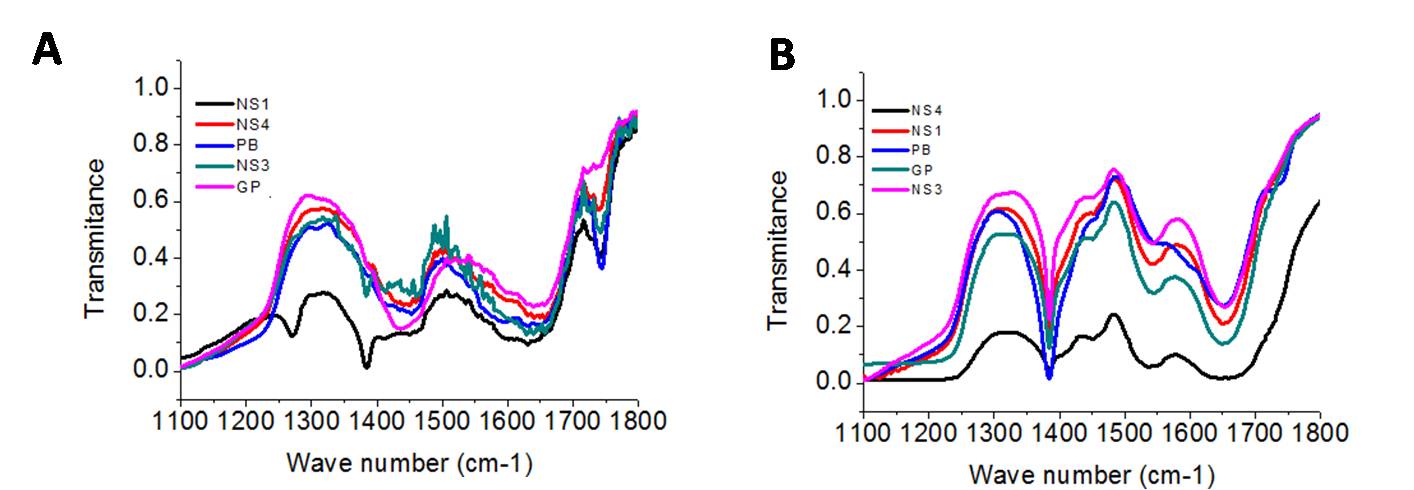
**

**Figure S1.** FT-IR spectra of five diatoms in the range of 1100-1800 cm^-1^ (A) at day 1 and (B) at day 30 of inoculation.

**
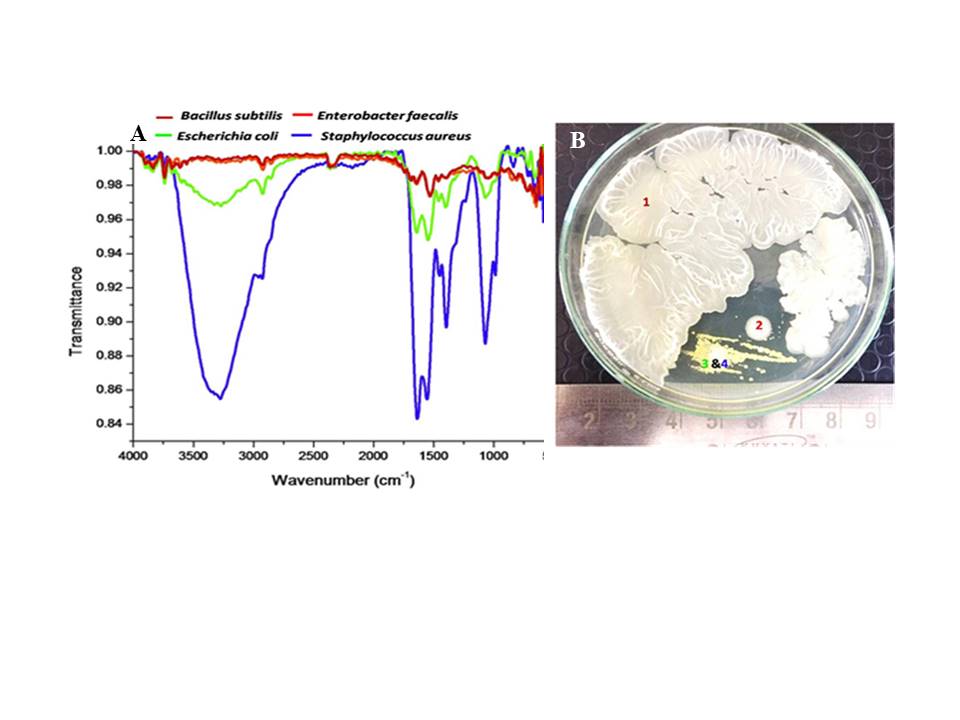
**

**Figure S2. A.** FT-IR spectra of four different bacteria *Bacillus subtilis*, *Enterobacter faecalis*, *Escherchia coli* and *Staphylococcus aureus* **(B)** Bacterial growth 1*. Bacillus subtilis*, 2. *Enterobacter faecalis*, 3. *Escherchia coli* and 4. *Staphylococcus aureus* in Luria bertani medium for 24 h.

**
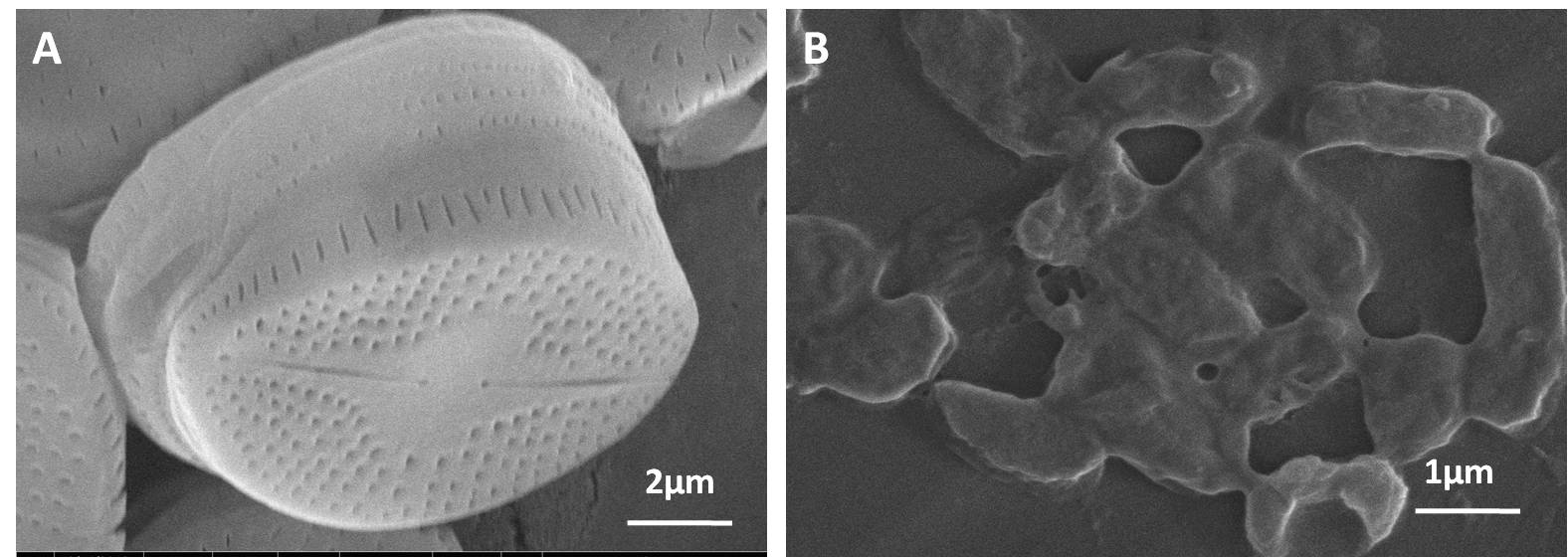
**

**Figure S3.** SEM images of control diatom *Nitzschia sp. 4* (A) and bacteria *Bacillus subtilis* (B).


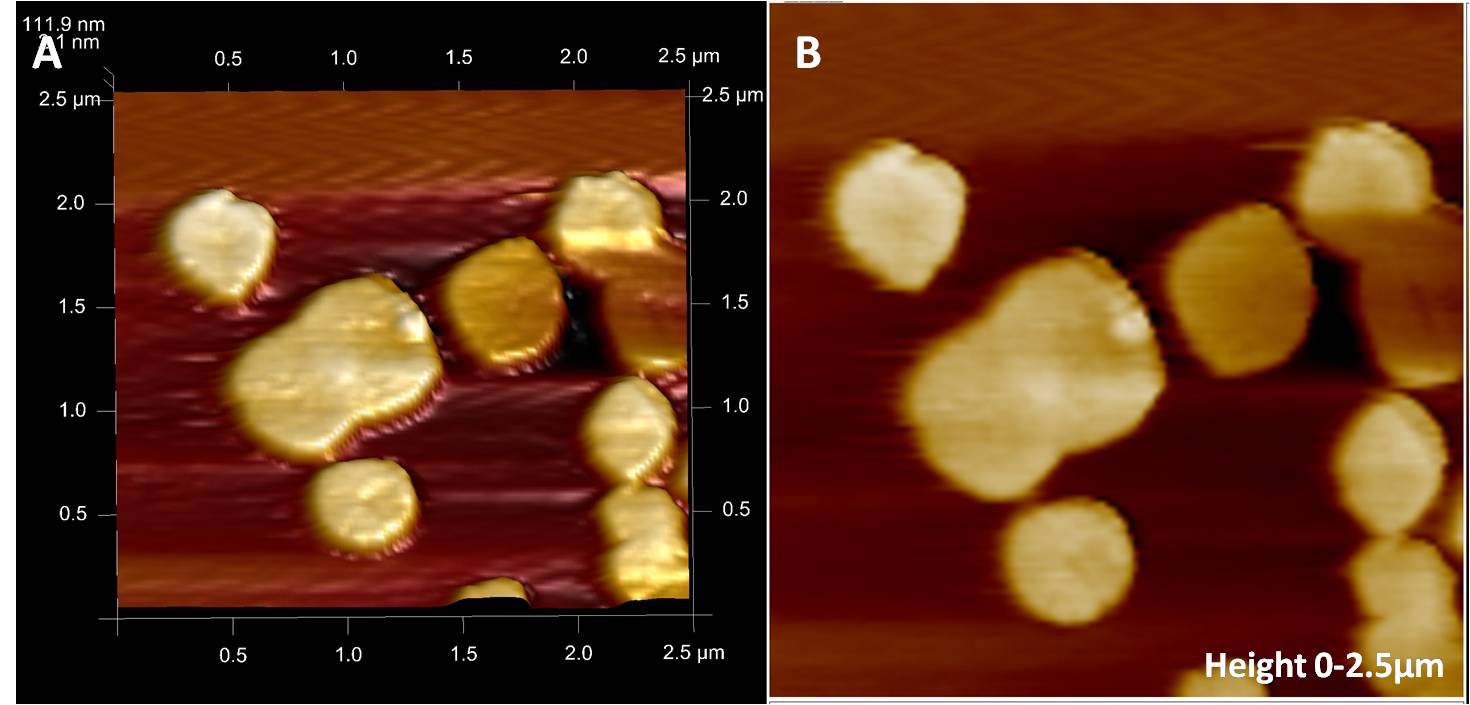


**Figure S4.** AFM images of *Bacillus subtilis* 3D (A) and 2D (B)

**
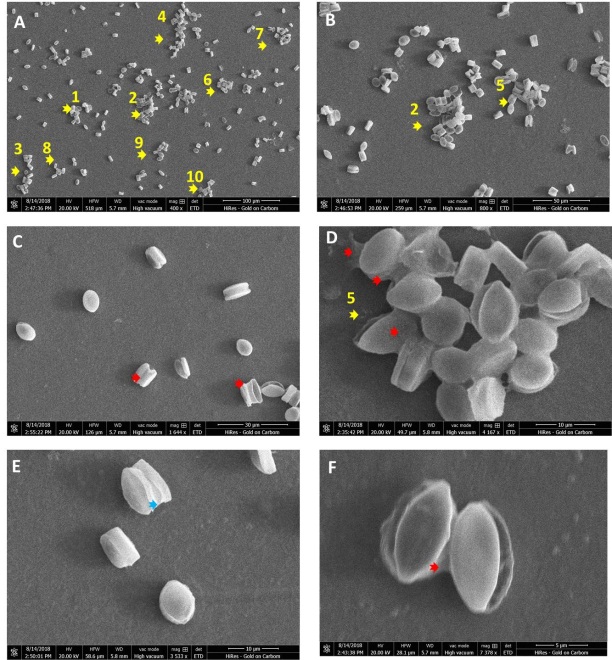
**

**Figure S5.** Diatoms on glass surface after 30 days

**
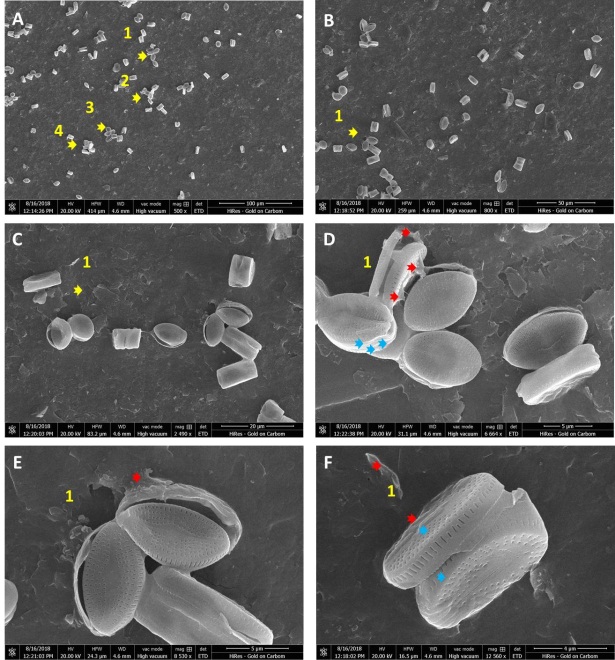
**

**Figure S6.** Diatoms on graphite surface after 30 days

**
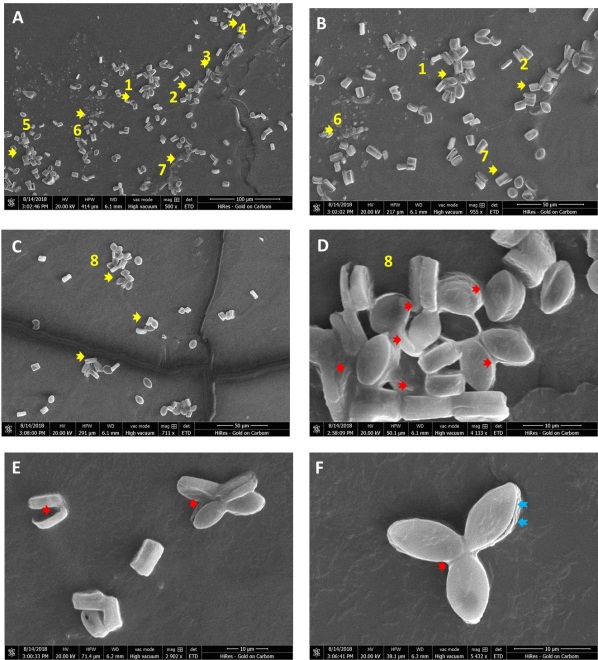
**

**Figure S7.** Diatoms on PDMS surface after 30 days

**
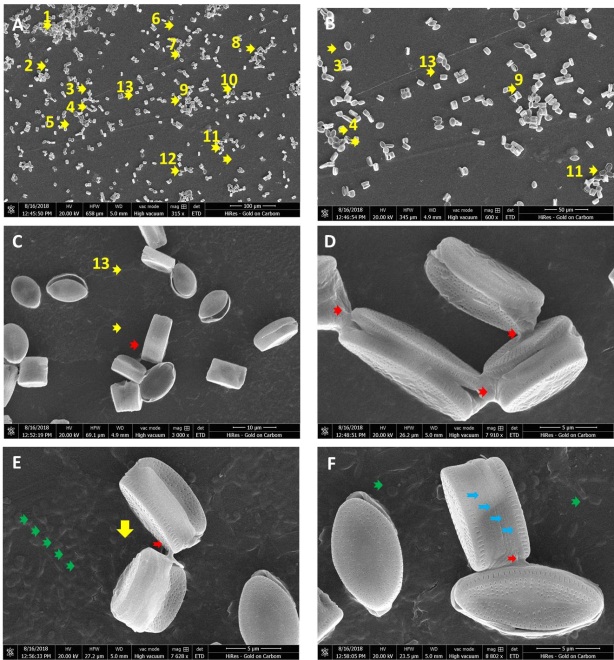
**

**Figure S8.** Diatoms on plastics surface after 30 days

**
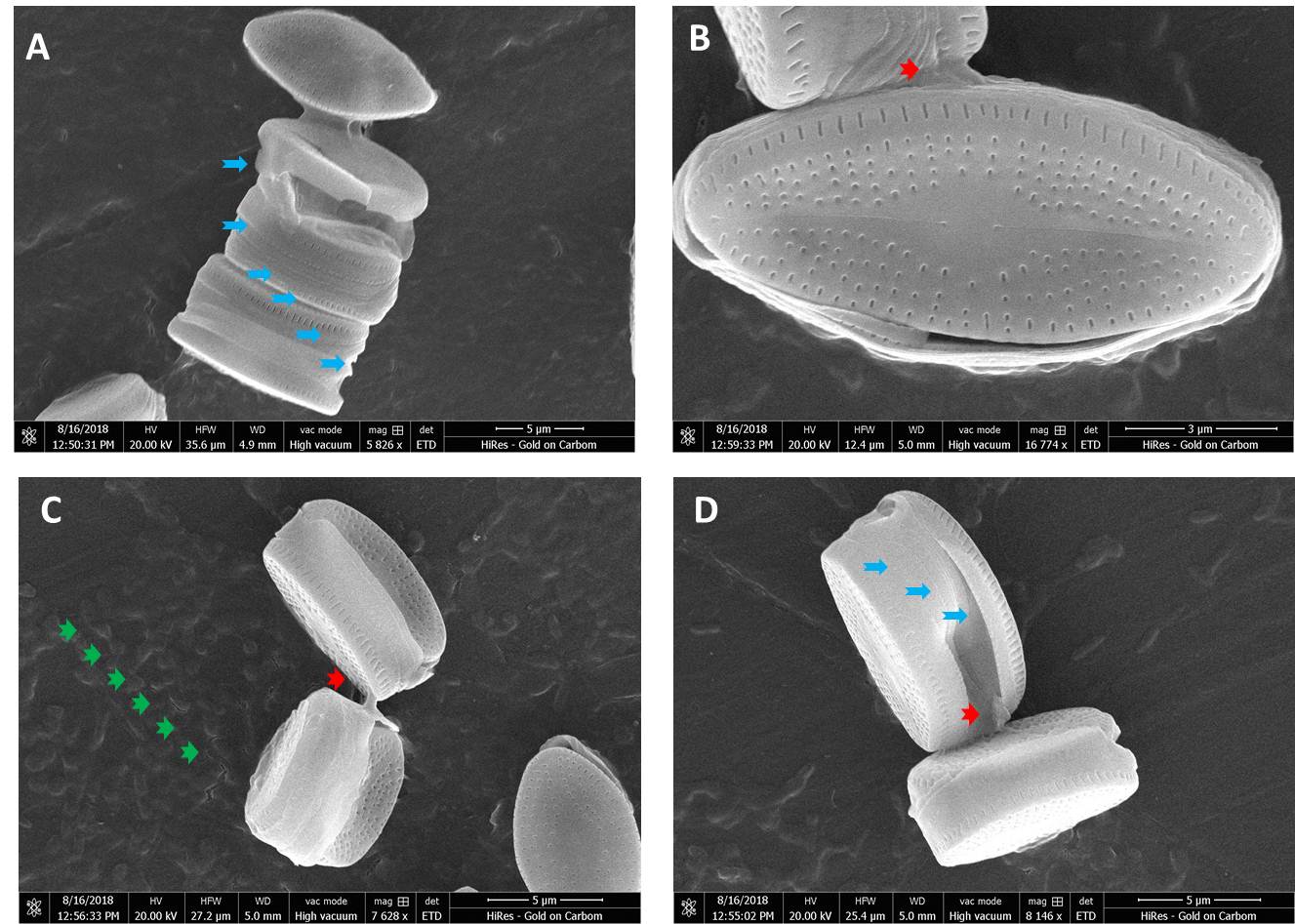
**

**Figure S9.** Diatoms on TiO_2_ surface after 30 days


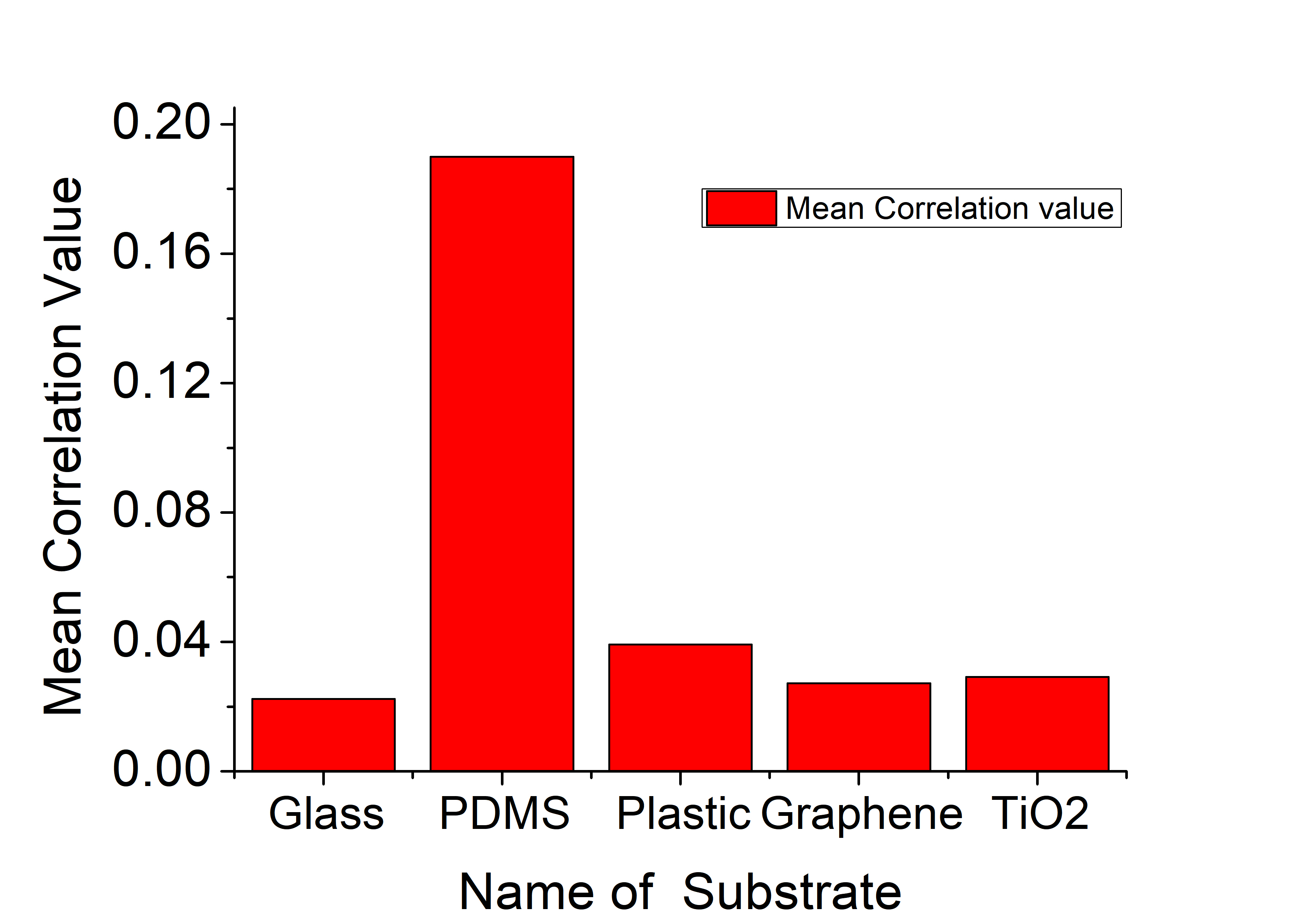


**Figure S10.** Mean correlation value of different substrates embellished on diatom *NS4*.
